# Supplementary figures and images for: A CRISPR-Cas9 screen reveals a role for WD repeat-containing protein 81 (WDR81) in the entry of late penetrating viruses
Source: PLoS Pathog. 2022 Mar 23;18(3):e1010398. doi: 10.1371/journal.ppat.1010398 (PMC8942271; doi:10.1371/journal.ppat.1010398)

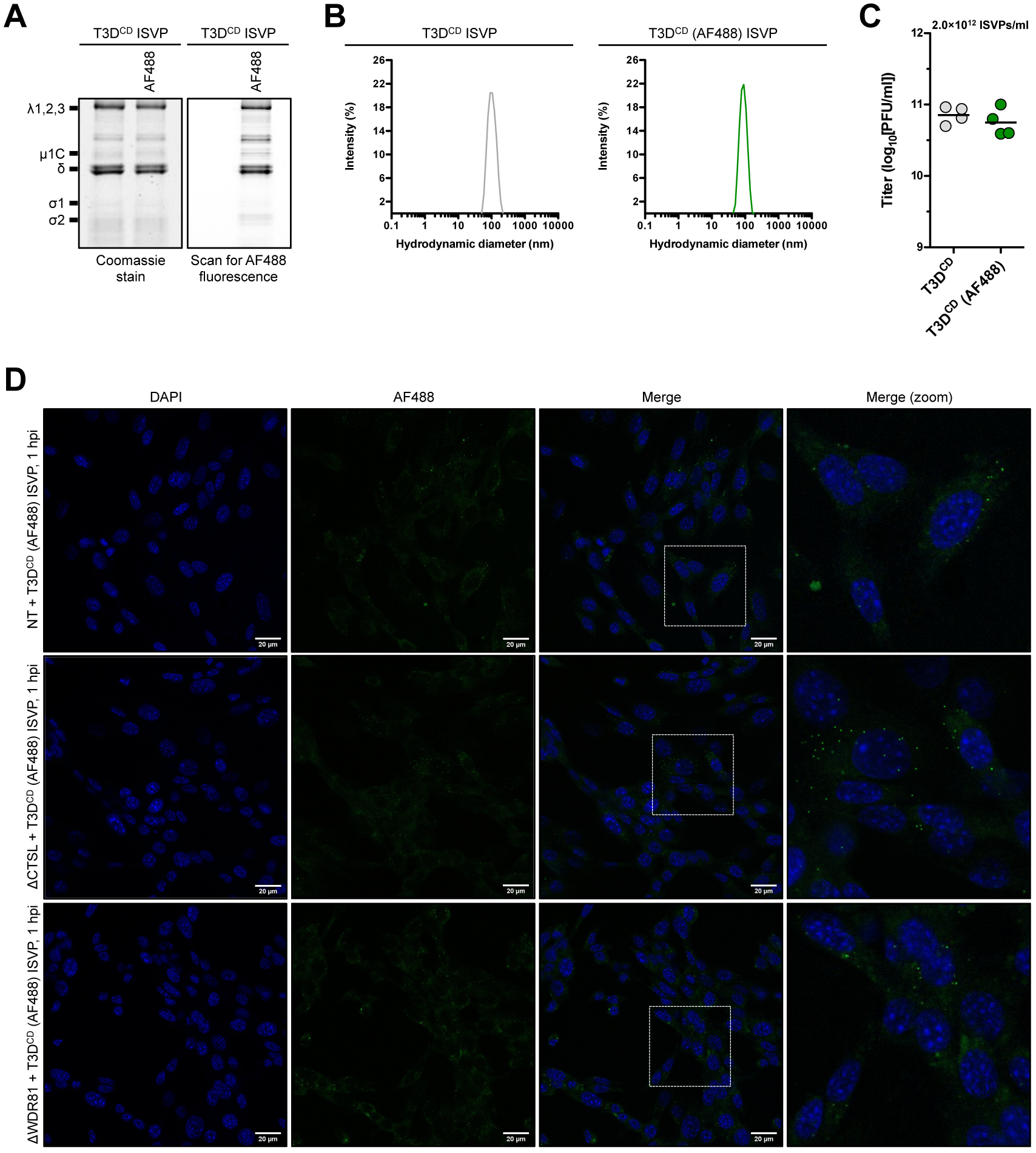

Supplement: S1 Fig — (A) Protein compositions. Unlabeled and AF488-labeled T3DCD ISVPs were analyzed by SDS-PAGE. The gel was Coomassie brilliant blue stained (left side panel) and scanned for AF488 fluorescence (right side panel). The migration of capsid proteins is indicated on the left. μ1δ resolves as δ [74] (n = 4 biological replicates; results for 1 representative experiment are shown). (B) Size distribution profiles. Unlabeled and AF488-labeled T3DCD ISVPs were analyzed by dynamic light scattering (n = 4 biological replicates; results from 1 representative experiment are shown). (C) Specific infectivity. The titers of unlabeled and AF488-labeled T3DCD ISVPs (2×10^12^ particles/ml) were determined by plaque assay. Horizontal bars indicate the means (n = 4 biological replicates). (D) Localization within an infected cell. NT, ΔCTSL, and ΔWDR81 cells were infected with T3DCD (AF488) at 10,000 ISVPs/cell. At 2 h post infection (hpi), the infected monolayers were fixed, stained with DAPI, mounted, and imaged by confocal microscopy. The images were obtained using a X63 oil-immersion objective and processed using ImageJ software [42]. The DAPI signal is false colored in blue, and the AF488 signal is false colored in green. The inset boxes in the ‘Merge’ column are expanded in the ‘Merge (zoom)’ column. The scale bars represent 20 μm (n = 3 biological replicates; results from 1 representative experiment are shown). (TIF) [file ppat.1010398.s001.tif]

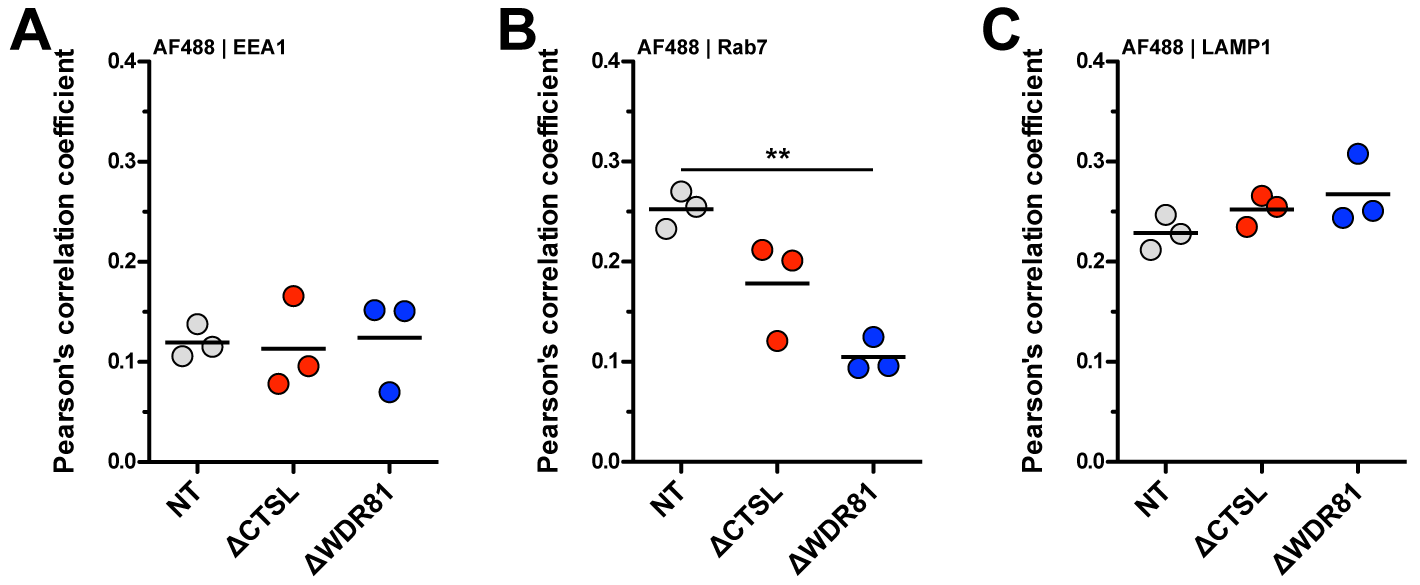

Supplement: S2 Fig — (A-C) NT, ΔCTSL, and ΔWDR81 cells were infected with T3DCD (AF488) at 10,000 virions/cell. At 2 h post infection (hpi), the infected monolayers were fixed, stained with an anti-EEA1, anti-Rab7, or anti-LAMP1 primary antibody and with DAPI, mounted, and imaged by confocal microscopy. The images were obtained using a X63 oil-immersion objective and processed using ImageJ software [42]. Pearson’s correlation coefficient between AF488 and EEA1 (A), Rab7 (B), or LAMP1 (C) was calculated using Just Another Colocalization Plugin (JACoP) [43]. Horizontal bars indicate the means; **, P ≤ 0.005 (n = 3 biological replicates). (TIF) [file ppat.1010398.s002.tif]
