# Supplementary material for: A CRISPR-Cas9 screen reveals a role for WD repeat-containing protein 81 (WDR81) in the entry of late penetrating viruses
Source: PLoS Pathog. 2022 Mar 23;18(3):e1010398. doi: 10.1371/journal.ppat.1010398 (PMC8942271; doi:10.1371/journal.ppat.1010398)
Supplement: Attachment — Submitted filename: CRISPR response to reviewer final.docx [file ppat.1010398.s004.docx]

**RESPONSE TO REVIEWERS**

**Major issues brought up by reviewers**

Reviewer #1

No issues were indicated

Reviewer #2

1. Experiments to determine the nature of the “dead-end compartment” would be very useful in interpreting the nature of the block in reovirus entry. Do these compartments contain lysosomal markers? Accumulation of early endosomal markers?

We have completed these experiments and we include these new data in Figs 7 and 8. We find that in absence of WDR81, some proportion of virus remains trapped in aberrant EEA1 and LAMP1 positive structures. We also find that Rab7 positive compartments are absent in absence of WDR81. We describe in the discussion why infection may be blocked in ΔWDR81 cells.

2. Experiments examining the location of WDR81 during reovirus entry would also aid interpretation of the potential role for WDR81 in the entry process (is it directly in the endosomal compartments containing virions, or does it exert effects through other means)?

WDR81 is known to is a cytoplasmic protein that localizes to early and late endosomes (PMID: 26783301 and PMID: PMID: 27126989). This location is consistent with the uptake pathway of reovirus which requires initial uptake of reovirus into early endosomes and then delivery into late endosomal compartments PMID: 22674975. We have clarified these points in the manuscript.

3. A replication time course, examining both reovirus transcript levels over time as well as virus particle production over time, would aid in clarifying the potential role for WDR81 – is it merely delaying entry and protein synthesis, or does it have a role in other processes that facilitate infection?

We have included experiments suggested that measure viral transcripts and infectious particle production over time. Based on the results of these experiments and from infection experiments initiated using in vitro generated ISVPs (Fig 5), we are confident that the effect of WDR81 is on entry.

Reviewer #3

1. Fig 5. The author speculated that virions or processed ISVP were accumulated in the late endosome (or the dead-end compartment) in WDR81-deficient cells. Co-localization analysis of viral particles and late endosome markers (such as Rab7) is recommended to ensure that the virions were stuck inside the endosome. It is also suggested to analyze the accumulation of virions in the cytoplasm (endosome) of NT and KO cells using electron microscopy.

This comment is similar to the comment made by reviewer 2. We have included data from new confocal microscopy experiments to identify the compartment in which virus particles are trapped in absence of WDR81. See Figures 7 and 8.

2. Fig 7. The author tested only two types of late penetrating viruses (reovirus and VSV-expressing Ebola G protein). Can VSV expressing ebolavirus glycoprotein be considered as a late-penetrating virus? References describing the characteristics of this virus should be included. A lot of viruses, including the dengue virus and influenza A virus, use late endosomes for cell entry. Since the title of this paper generalized that WDR81 is involved in cell entry of late penetrating virus, the test using other viruses is recommended.

VSV-expressing ebolavirus GP has been extensively used to characterize Ebolavirus entry. Available data indicate that this virus uses entry pathways that identical to those used by Ebolavirus (for example VSV-EboV GP and native EBOV both depend on cathepsins and NPC1). Thus, this virus is good BSL2 surrogate for a late penetrating virus. It is also an excellent tool because both our and many previous studies indicate distinct entry requirements for VSV and VSV-Ebo-GP. References describing these statements are now included in the manuscript.

We currently do not have expertise for, access to, or IBC approval for use of influenza or Dengue. Thus, these studies are likely to take longer and we feel beyond the scope of our work. During revision we became aware of another manuscript posted on bioRxiv (https://doi.org/10.1101/2021.08.06.455441) which indicates that influenza virus infection is not impacted by the absence of WDR81. We think that published work on the requirement of WDR81 for coronavirus infection support our conclusion that many late penetrating viruses require WDR81. We have tempered our language in the discussion to indicate the speculative aspect of our conclusion.

**Minor issues brought up by reviewers**

Reviewer #1

Several text changes were suggested.

We have incorporated most of these suggestions.

Reviewer #2

1. The CRISPR-engineered mutation causes a premature stop codon at residue 1338 of 1934, so over 2/3 of the protein is still encoded. Clearly, the protein is not functional to support reovirus infection, but what is known about the structure of the protein that might limit its activity following truncation? Are transmembrane domains missing? More information about the known domain architecture/structure of the protein would be helpful. Additionally, an immunoblot for the protein (I believe a polyclonal antiserum is commercially available?) would help to understand whether a truncated product (dominant negative?) is produced or whether the truncation leads to an unstable/abortive product.

Not much is known about the structure-function relationships in mammalian WDR81. Based on our complementation experiments, we do not believe the knockout is dominant negative. We did not attempt to detect the truncated form of the protein in our cells.

2. Data on virus replication is presented as raw titers, not virus yield. Presenting yield instead of/in addition to virus titers may help interpret the relative impact of WDR81 on the system. Relatedly, the authors claim on line 538 that WDR81 is “required for replication” – clearly, some replication is happening, though not as efficiently as in wild type cells. (As an aside, this is, in itself, interesting – is the virus able to replicate to wild type titers in some small subset of cells that it can gain cytoplasmic access to, or is replication just less efficient or delayed in all cells, leading to the decrease in yield?).

Our data in Figure 2 indicate that attachment of virus to wild-type and ΔWDR81 cells is equivalent. Therefore, we expect that virus yields in these two cells types defer by 1.5 log_10_ (a 97% reduction) in yield. Based on our infectivity measurements using indirect immunofluorescence, we think that very few cells are infected even in absence of WDR81. Additional virus growth analysis over a time course indicates that reovirus replicates to a greater extent in ΔWDR81 cells in comparison to ΔCTSL cells (See Figure 3). Nonetheless, reovirus replication is still significantly lower in ΔWDR81 cells than in NT cells and never catches up. We have modified our text to indicate that WDR81 is required for efficient replication.

3. Similarly, the experiments with VSV-EBO GP are not conducted to a time when replication plateaus – is replication merely delayed in the WDR81-deficient cells, or will it only infect a subset of the total (clearly the effect in the CTSL-knockouts is more significant, but it is hard to tell where the curve in the WDR81 cells would end up if allowed to progress further).

Our live cell imaging experiments and titer data would suggest that the need for WDR81 is not absolute for VSV-EBO-GP. While we see a difference in the number of infected cells, the titers though statistically different do not rise to the level of 1 log_10_ difference that we consider biologically relevant. The text is modified to indicate that WDR81 is required for replication with optimal kinetics.

Reviewer #3:

1. Fig 2. What if the endocytosis inhibitor (such as dynasore) is used? Do the NT cells show similar infectivity to WDR81 deficient cells? What is the balance between cathepsin B and L? Does cathepsin B compensate the function of cathepsin L to induce mu1 cleavage in CTSL deficient cells? In Fig 2A, it was mentioned that the cell viability was checked at 24h and 48h post-infection in the figure legend. However, the analysis at 24h post-infection result is nowhere to be found. Please confirm the results.

We did not complete experiments to determine the effect of an endocytosis inhibitor (dynasore). Reovirus can be taken up into cells via multiple uptake pathways (PMID: 22973022) and it is not known which uptake pathway is predominantly used in MEFs. Based on evidence that virions are disassembled to ISVPs in absence of WDR81, we expect that particles are endocytosed into a compartment containing active cathepsin L. Therefore, blocking virus uptake will be act upstream of either WDR81 and cathepsin L.

Initial characterization of a role for cathepsins in reovirus infection indicated that cathepsin B and L can both function to disassemble reovirus particle (PMID: 11986312). That study also stated that inhibition of both cathepsins in necessary to block infection. In the given time frame of our experiments, we did not observe any μ1 cleavage in absence of cathepsin L. We do not know if low level μ1 cleavage mediated by cathepsin B or other proteases is not detectable or whether it does not take place in our cells. We note that cathepsin B was not identified in our CRISPR screen.

We only showed cell viability data at 48 h. This information is corrected in the legend.

2. Figs 2 and 3. In Figs 2G-H, it was revealed that the viral titer in ΔWDR81 cells was decreased in accordance with its infectivity. However, in Figs 3C-D, the reovirus infectivity of WDR81-complemented cells was still decreased, but the viral titer of WDR81-complemented cells was the same as that of NT-empty cells. I am intrigued whether the ΔWDR81 cells inhibit the reovirus infection completely or just delay the replication. Thus, I would suggest multiple step reovirus growth assay to address this. In addition, what is the reason for using human WDR81 instead of murine WDR81? The homology between those two genes is pretty high, however, infectivity in WDR81-complemented cells is still different from that in NT cells, hence it is not ideal complementation unless there is any explanation. Please provide any reasoning regarding this decision.

We have included a time course measuring viral progeny production. Progeny production over multiple cycles remains low in absence of WDR81. Based on our infectivity measurements based on indirect immunofluorescence, we think that very few cells are infected in absence of WDR81.

We used human WDR81 because a tagged form of this protein was previously characterized. We adopted this solution to avoid issues related to commercially available WDR81 sera that can detect murine WDR81. While infectivity was not fully restored, we observed a significant recovery to the extent that ~ 50% complemented cells were now infectable. This level of infection is consistent with our titer data where a two-fold change in titer would not score as detectable or statistically significant because it will be within the same order of magnitude.

3. Fig 5. Why is the virus not accumulated in the late endosome in CTSL deficient cells? As mentioned in the manuscript, the virions are not able to be converted to ISVP in the CTSL deficient cells, resulting in the failure of viral penetration. The author should give a discussion on that part.

In new experiments we have included, we find virus in EEA1, Rab7 and LAMP1 positive compartments in both NT and ΔCTSL cells.

4. Line 85. .. is a segmented double-stranded RNA (dsRNA) virus..

We have made this correction

5. The author identified several genes including WDR81, WDR91, and CTSL that were required for the reovirus infection by CRISPR-Cas9 screening and focused on the role of WDR81 (and CTSL) in reovirus infection. The author also mentioned that WDR81 and its binding partner WDR91 play an important role in endosomal maturation. Thus, the author might want to include the data indicating the importance between WDR81 and WDR91 interaction in reovirus infection if possible.

Not much more is known about the interaction between mammalian WDR81 and WDR91 than we have indicated in the paper.
