# Supplementary material for: A CRISPR-Cas9 screen reveals a role for WD repeat-containing protein 81 (WDR81) in the entry of late penetrating viruses
Source: PLoS Pathog. 2022 Mar 23;18(3):e1010398. doi: 10.1371/journal.ppat.1010398 (PMC8942271; doi:10.1371/journal.ppat.1010398)
Supplement: Attachment — Submitted filename: Dear PLoS Pathogens Editors.docx [file ppat.1010398.s005.docx]

Dear PLoS Pathogens Editors,

Thank you for reviewing our revised manuscript "A CRISPR-Cas9 screen reveals a role for WD repeat-containing protein 81 (WDR81) in the entry of late penetrating viruses".

We are pleased to hear that you found most of our previous revisions satisfactory and that you are likely to accept our manuscript if we include quantitation of the data in Figure 7 and 8, and revise our manuscript accordingly.

In this new version, we have quantified our colocalization data presented in Figure 7 and 8. These data are now included as Figure S2. Our data support the notion that some proportion of virus particles appear colocalize with EEA1 and LAMP1 in all conditions. The quantified data suggest that that proportion is equivalent. The quantitation also demonstrate a lower localization with Rab7 in absence of WDR81, similar to what we had concluded from subjective observations.

In this revised version, we have included the a new figure legend, and the method used for quantitation. We made small adjustments to the results section to include the data from quantitation. The overall conclusions remain unchanged.

We are grateful to the reviewers and editors for their insightful comments, and we think that the revisions we have made based on their efforts improve the quality of our paper. Thank you very much for your consideration.

Sincerely,

Pranav Danthi

Professor of Biology
